# Effects of* Bacillus* Serine Proteases on the Bacterial Biofilms

**DOI:** 10.1155/2017/8525912

**Published:** 2017-08-21

**Authors:** Olga Mitrofanova, Ayslu Mardanova, Vladimir Evtugyn, Lydia Bogomolnaya, Margarita Sharipova

**Affiliations:** ^1^Institute of Fundamental Medicine and Biology, Kazan Federal University, Kazan, Russia; ^2^Texas A&M University Health Science Center, Bryan, TX, USA

## Abstract

*Serratia marcescens* is an emerging opportunistic pathogen responsible for many hospital-acquired infections including catheter-associated bacteremia and urinary tract and respiratory tract infections. Biofilm formation is one of the mechanisms employed by* S. marcescens* to increase its virulence and pathogenicity. Here, we have investigated the main steps of the biofilm formation by* S. marcescens* SR 41-8000. It was found that the biofilm growth is stimulated by the nutrient-rich environment. The time-course experiments showed that* S. marcescens* cells adhere to the surface of the catheter and start to produce extracellular polymeric substances (EPS) within the first 2 days of growth. After 7 days,* S. marcescens* biofilms maturate and consist of bacterial cells embedded in a self-produced matrix of hydrated EPS. In this study, the effect of* Bacillus pumilus* 3-19 proteolytic enzymes on the structure of 7-day-old* S. marcescens* biofilms was examined. Using quantitative methods and scanning electron microscopy for the detection of biofilm, we demonstrated a high efficacy of subtilisin-like protease and glutamyl endopeptidase in biofilm removal. Enzymatic treatment resulted in the degradation of the EPS components and significant eradication of the biofilms.

## 1. Introduction

It has been established that most bacteria do not live as free-floating planktonic cells but instead they aggregate in microbial communities called “biofilms” [1]. Even though biofilms formed by different bacterial species may vary a lot, they still share some common features. Biofilms are tightly packed aggregates of microbial cells attached to the surface and surrounded by a self-produced extracellular matrix [2]. Biofilm matrix is comprised of different macromolecules known as extracellular polymeric substances (EPS). EPS serve as a scaffold for the tertiary structure of the biofilm, allow cell-to-cell communication, and are responsible for the adhesion to surfaces [3]. Bacterial cells embedded in a polymeric protective matrix are able to survive in unfavorable environment conditions and are much less susceptible to the antimicrobial agents. Most of the antibiotics cannot penetrate biofilms and are inactivated by the extracellular matrix [4, 5]. In most biofilms, matrix accounts for about 90% of the total biomass, whereas bacteria account for the remaining 10% [6]. EPS consist of a wide range of biopolymers such as polysaccharides, proteins, lipids, and nucleic acids [7]. Until recently, polysaccharides were considered the major component of the biofilms [8]. However, new studies suggest that proteins also play a crucial role in the biofilm formation. They facilitate microbial adhesion to the surfaces and ensure the mechanical stability of the biofilms [9, 10]. Despite numerous attempts to develop a strategy for the biofilm eradication, there is still a need for the reliable methods aimed at preventing and combating biofilms.

Enzymatic degradation of the EPS components is one of the attractive strategies for the biofilm removal [11, 12]. Recent reports indicated that various microbial enzymes may be used as potential matrix-degrading therapeutic agents. For instance, dispersin B produced by* Actinobacillus actinomycetemcomitans* was found to be effective against polysaccharide adhesins within the biofilms of different bacteria [13]. Both* Pseudomonas aeruginosa* LasB elastase and Esp serine protease secreted by* Staphylococcus epidermidis* emerge as major antibiofilm proteases that inhibit* S. aureus* biofilm formation and detach preexisting biofilms [14, 15]. Application of* Serratia marcescens* metalloprotease serratiopeptidase resulted in the suppression of the biofilm formation by* Listeria monocytogenes* [16]. In addition to these above-mentioned bacterial species,* Bacillus* is also known to produce an array of extracellular proteolytic enzymes.* Bacillus* proteases have high stability and low pathogenicity and can be easily purified and obtained in industrial quantities. These features make them promising matrix-degrading agents for combating bacterial biofilms.

*Serratia marcescens* is a well-recognized opportunistic and nosocomial pathogen responsible for many hospital-acquired infections. It accounts for 1-2% of the nosocomial infections related to the respiratory tract, the urinary tract, surgical wounds, and soft tissues. In most cases,* S. marcescens* is a serious threat to the patients during hospitalization, placement of intravenous catheters, intraperitoneal catheters, and urinary catheters, and prior instrumentation of the respiratory tract [17, 18]. Moreover, measuring devices such as urinometer used in the health care facilities were also found to be colonized with* S. marcescens* [19]. Examination of clinically isolated* S. marcescens* strains revealed that most of them possess fimbriae capable of adherence to the human uroepithelial cells and considered to be a colonization factor in the urinary tract infections [20].* S. marcescens* is resistant to many antibiotics traditionally used to eradicate bacterial infections, including *β*-lactams and aminoglycosides [21, 22]. Its motility and ability to form flagellated swarmer cells as well as the biofilm formation are important virulence/pathogenicity factors [23]. Taking into consideration the fact that* S. marcescens* infections could be transmitted through hand-to-hand contact by medical personnel and could be inoculated directly to the catheterized patient, there is a growing need to develop new ways aimed at the pathogen elimination.

In the present study, we evaluated the efficacy of* Bacillus* proteases in the biofilm dispersal using* S. marcescens* SR 41-8000 biofilms as a model.

## 2. Materials and Methods

### 2.1. Bacterial Strains, Media, and Growth

*S. marcescens* SR 41-8000 [24] was routinely grown at 37°C with a constant agitation at 200 rpm overnight in Luria-Bertani (LB) broth containing (per L) 10 g tryptone, 5 g yeast extract, and 5 g NaCl. To study the biofilm formation, bacteria were grown in 3 different media, LB broth, minimal broth Davis (MBD) containing (per L) 7 g K_2_HPO_4_, 2 g KH_2_PO_4_, 0.5 g Na_3_C_6_H_5_O_7_, 0.1 g MgSO_4_, and 1 g (NH_4_)_2_SO_4_ supplemented with 0.5% glucose, and Mueller-Hinton (MH) broth (Becton Dickinson, USA), under the same conditions for 7 days at 30°C.

### 2.2. Biofilm Formation

Bacteria were grown in 12-well polystyrene tissue culture treated plates (Corning Costar, USA) as described by Christensen et al. [25] with some modification. The overnight cultures were diluted to OD600 = 0.2 with fresh media and 3 ml of the bacterial suspension was transferred into each well of the tissue culture plate. Negative control wells contained an equal volume of the sterile medium. Plates were incubated at 30°C for 7 days and biofilm formation was monitored daily. Each day, a set of wells was used for the biofilm formation evaluation. Bacterial cultures were removed by aspiration; wells were washed three times with 3 ml of phosphate-buffered saline (PBS) (Sigma, USA), pH 7.4. Biofilms were air-dried and stained with 3 ml of 0.1% crystal violet (Dia-M, Russia) for 20 min. Subsequently, the dye was removed and the wells were washed 5 times with sterile PBS. Wells were allowed to dry and resulting stained biofilms were solubilized with 3 ml of ethanol. To evaluate the biofilm formation, 200 *μ*l from each well was used to measure the optical density (OD) at 595 nm using microtiter-plate reader (iMark Microplate Absorbance Reader, Bio-Rad, Japan). Experiments were performed in triplicate and repeated on three different occasions. Evaluation of the biofilm formation was performed using the criteria of Stepanović et al. [26], where the defined cut-off value separates biofilm-producing strains from non-biofilm-producing strains.

### 2.3. Sample Preparation for Scanning Electron Microscopy (SEM)

*S. marcescens* biofilm formation on the catheter surface was evaluated by scanning electron microscopy. Biofilms were grown on the fragments of polyvinylchloride urethral catheter (surface area: 1 sq. cm). Catheters were sterilized with 96% ethanol, rinsed with distilled water, and autoclaved. Each catheter fragment was placed in a well of the 12-well tissue culture plate filled with diluted bacterial cultures as described above. Wells filled with sterile media served as a negative control. For the SEM sample, preparation catheters were washed three times with 3 ml of PBS to remove nonadherent cells. Subsequently, catheters were fixed in 2.5% glutaraldehyde/PBS, pH 7.4 (Sigma, USA), at room temperature with constant agitation for 5 hours. Next, catheters were washed three times with PBS and dehydrated in 30%, 40%, 50%, 70%, 80%, and 90% ethanol for 15 min at each concentration and three times in 96% ethanol for 15 min each. After drying, catheters were coated with gold palladium and then imaged using MERLIN scanning electron microscope (Carl Zeiss, Germany) in a high vacuum mode at 10 kV. Scanning electron micrographs of the biofilms were taken at 5,000x, 10,000x, and 20,000x magnification.

### 2.4. Microscopic Evaluation of Planktonic Cells by SEM

Bacteria were grown at 37°C with a constant agitation at 200 rpm overnight. 1 ml of cell suspension was placed into a sterile 1.5 ml tube and centrifuged for 15 min at 4,000 rpm at 15°C. Pelleted cells were washed in PBS twice. After centrifugation, collected cells were resuspended in 1% glutaraldehyde/PBS, pH 7.4, and incubated overnight at 22°C. Then cells were rinsed three times with PBS and dehydrated in ethanol at the concentrations of 30%, 40%, 50%, 70%, 80%, and 90% (for 15 min each). Next, cells were suspended in 96% ethanol and centrifuged for 20 min at 5,000 rpm. The procedure was repeated three times. The resulting cell suspension was spread over the glass coverslip surface and dried. After the drying, glass coverslips were coated with gold palladium (Quorum Q150T ES vacuum coater, UK) and imaged using MERLIN scanning electron microscope (Carl Zeiss, Germany) in a high vacuum mode at 15 kV. Scanning electron micrographs of planktonic cells were taken at 10,000x magnification.

### 2.5. Confocal Laser Scanning Microscopy of the Biofilms

The 2-day-old and 4-day-old biofilms formed in the tissue culture treated Petri dishes (35 mm × 10 mm) (Eppendorf, Germany) were washed twice with PBS. Biofilm cell viability was examined using live/dead staining for 20 min at room temperature in the dark, where DiOC_6_(3) (green fluorescent) and propidium iodide (PI) (red fluorescent) dyes (Sigma, USA) were used as live stain and dead stain, respectively. Images were visualized by confocal laser scanning microscopy (CLSM) using inverted Carl Zeiss LSM 780 microscope (Carl Zeiss, Germany). PI fluorescence was measured at 633 nm and DiOC_6_(3) fluorescence was measured at 488 nm.

### 2.6. Congo Red Binding Assay

Biofilm production was detected in Congo red (CR) binding assay [27]. Bacteria were grown on the LB agar plates containing 25 *μ*g/ml of CR dye at 30°C. Plates were photographed after 24 h and 72 h. An increase in CR absorption with the time was visually detected and indicated biofilm production. For the quantitative CR binding assay, bacteria were grown on the LB agar plates for 7 days at 30°C. Every 24 h colonies were scraped off and suspended in 0.9% NaCl. Triple dilutions were performed, and the bacterial concentration was quantified by measuring OD600 against a saline background. Bacteria were then pelleted by centrifugation for 10 min at 14,000 rpm. A 0.002% solution of CR (in 0.9% NaCl) was prepared, and its OD500 was measured against a saline background. Then bacteria were resuspended in 1 ml of CR solution dye and left for 10 min of binding at room temperature, followed by a second centrifugation (under the same conditions). The dye solution was recovered, and the reduction in optical density was determined by measuring OD500 [28].

### 2.7. Proteolytic Activity of Enzymes

Proteolytic activity of the enzymes was determined using Z-Ala-Ala-Leu-pNA as a substrate for subtilisin-like protease as described by Voyushina et al. [29] and synthetic Z-Glu-pNA as a substrate for glutamyl endopeptidase as described by Leshchinskaya et al. [30]. The activity unit was defined as the amount of the enzyme able to produce 1 *μ*mol of* p*-nitroaniline (pNA) per min from Z-Ala-Ala-Leu-pNA or Z-Glu-pNA under the specific conditions.

### 2.8. Protein Extraction from the Catheter Biofilm

Catheters with biofilms were transferred to tubes with 1 ml of MH broth, incubated on ice for 5 min, mixed vigorously for 30 sec, and placed back on ice for additional 10 min. After repeating these steps twice, catheters were treated by ultrasound at 40 W (Vibra-Cell 505 ultrasonic processor, USA) for 30 sec five times at intervals of 1 min in an ice bath. Resulting cell lysates were mixed with ice-cold acetone (1 : 7, v/v). After centrifugation (14,000 rpm, 12°C, 40 min), the resulting protein pellet was dried and solubilized in PBS. Total protein concentration was determined using Bradford assay [31] based on the bovine serum albumin (BSA) (Dia-M, Russia) standard curve.

### 2.9. Statistical Analysis

Microsoft Excel was used to perform statistical analysis where appropriate. Values are the mean ± SD of these experiments. Significance between experimental values was assessed by Student's *t*-test.

## 3. Results and Discussion

### 3.1. Rich Media Promotes Biofilm Formation by* S. marcescens* SR 41-8000 Cells

**Figure 1:**
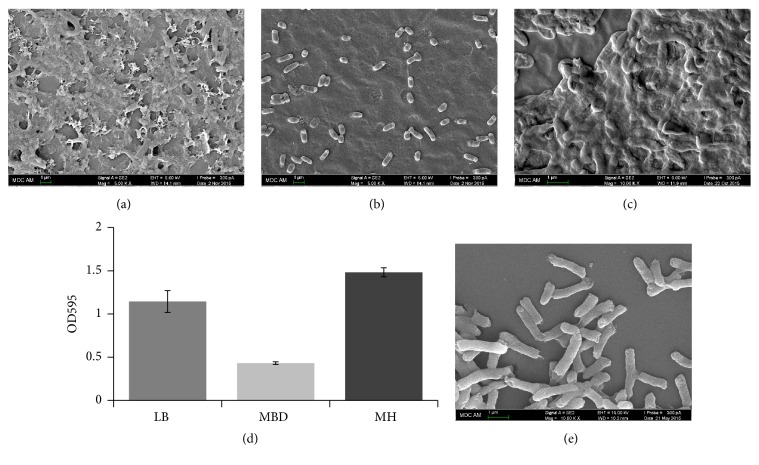
*S. marcescens* SR 41-8000 biofilm formation on catheters incubated in different growth media. ((a)–(c)) Catheters were incubated at 30°C for 6 days, fixed with 2.5% glutaraldehyde, and photographed at 5,000x magnification. Bars: 1 *μ*m. (a) Biofilms grown in LB broth. (b) Biofilms grown in MBD/0.5% glucose medium. (c) Biofilms grown in MH broth. (d) Biofilm formation in LB broth, MBD/0.5% glucose medium, and MH broth evaluated in crystal violet assay. (e) Morphology of free-living* S. marcescens* cells. Sample preparation of planktonic cells was performed as described in Section 2. Scanning electron microscopy image of the free-living* S. marcescens* cells was taken at 10,000x magnification. Bars: 1 *μ*m. Experiments were performed in triplicate and repeated three times. Error bars represent standard deviations.

The attachment of bacteria and biofilm formation depend largely on different environmental factors such as pH, temperature, O_2_ levels, and nutrient conditions [32]. It has been previously shown that bacteria growing in a high-nutrient medium resulted in the formation of thicker and more densely packed biofilms [33]. Preliminary experiments demonstrated that* S. marcescens* SR 41-8000 strain shows a better formation of the biofilms at a temperature of 30°C (data not shown). To evaluate the ability of* S. marcescens* to form biofilms in different media, we used nutrient-rich LB broth and MH broth as well as minimal defined medium MBD supplemented with 0.5% glucose. As expected, SEM images of the 6-day-old biofilms formed on the catheters revealed strong dependence of the biofilm formation on the growth media. The formation of a slimy biofilm layer with bacterial cells embedded in an extracellular matrix was favored by high-nutrient conditions present in LB and MH broth (Figures 1(a) and 1(c)). In a striking contrast, under the low-nutrient conditions, biofilm formation was very inefficient and only single cells were present (Figure 1(b)). Morphology of these cells closely resembled the morphology of the free-living cells (Figure 1(e)). Cells can be described as long rod-shaped bacteria, 1.2–2.3 *μ*m in length and 0.3–0.5 *μ*m in width. These results are in a good agreement with the previously obtained data that a switch between the filamentous biofilm morphology and microcolony type of the biofilm is controlled by the nutrient enrichment and limitation [34]. Next, we used the same growth conditions to allow the biofilm formation on the well surface of the tissue culture treated plates. After 6 days of growth, biofilm formation was evaluated in crystal violet assay. In concordance with the SEM results (Figures 1(a)–1(c)), quantification of biofilms showed that both LB and MH broth promote* S. marcescens* biofilm development and the latter media results in a maximum accumulation of the biofilms (Figure 1(d)). Therefore, MH broth was used in all subsequent experiments.

### 3.2. Scanning Electron Microscopy and Fluorescent Microscopy Show the Complexity of* S. marcescens* Biofilms

**Figure 2:**
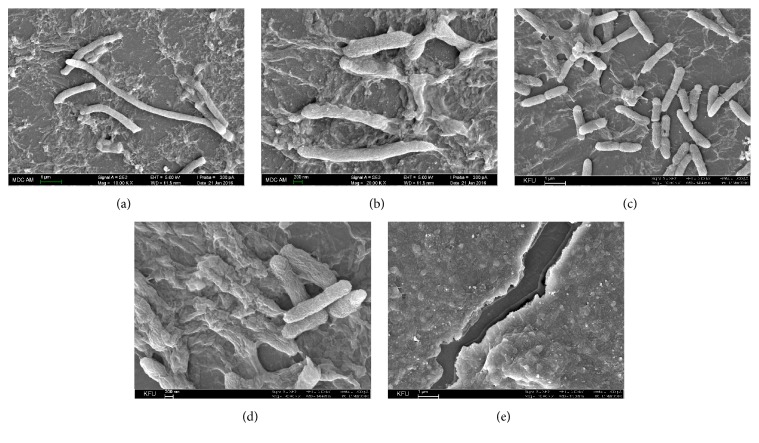
Morphological changes of* S. marcescens* cells during biofilm formation. Initial steps of the biofilm formation result in the appearance of elongated cells on the catheter surface after 3 days of incubation in MH broth at 30°C. Images were taken at 10,000x (a) and 20,000x (b) magnification. Bars are 1 *μ*m and 200 nm, respectively. Extracellular matrix production by* S. marcescens* cells analyzed after 3 (c), 4 (d), and 7 (e) days of incubation progresses over the time. Images were taken at 10,000x (c, e) and 20,000x (d) magnification. Bars are 1 *μ*m and 200 nm, respectively.

SEM provides us with the most detailed, in-depth visual analysis of the biofilm structural components. Biofilm formation inevitably results in the differentiation of cells to various phenotypic states [35]. During the switch from free-living planktonic cells to a biofilm community,* S. marcescens* cells undergo phenotypic changes resulting in the formation of elongated swarmer cells, which is consistent with previous findings [23] (Figures 2(a) and 2(b)). The swarmer cells promote migration of cells along the surface and facilitate the process of biofilm development [36]. As the biofilm formation progresses, bacterial cells become surrounded by a cohesive mass of the EPS that interconnect and tightly hold biofilm cells on the surface (Figures 2(c)–2(e)). The extracellular matrix is composed of various filaments, fibers, fimbriae, and amyloid-like structures that anchor bacteria on the surface and allow their irreversible attachment for further growth and maturation of the biofilm. Fimbriae, one of the major virulence factors of* S. marcescens,* consist of adhesins mediating the adherence to the biological surfaces. In addition,* S. marcescens* produces extracellular proteases, nucleases, lipases, chitinases, and hemolysin which act as pathogenicity factors and constitute a mucoid mass of the biofilm matrix.

Next, we stained* S. marcescens* biofilms with live/dead stain to visualize their structure at different stages. Fluorescent staining combined with confocal laser scanning microscopy (CLSM) allows researchers to carry out direct microscopic imaging of the biofilm heterogeneity and inspect cell localization and biofilm thickness. We stained biofilms with two fluorescent dyes: propidium iodide (PI) (stain for dead cells) and DiOC_6_(3) (stain for live cells). DiOC_6_(3) penetrates bacterial cells and accumulates in the cytoplasm, resulting in the green signal, while propidium iodine selectively stains cells with compromised membrane integrity which appear red.

**Figure 3:**
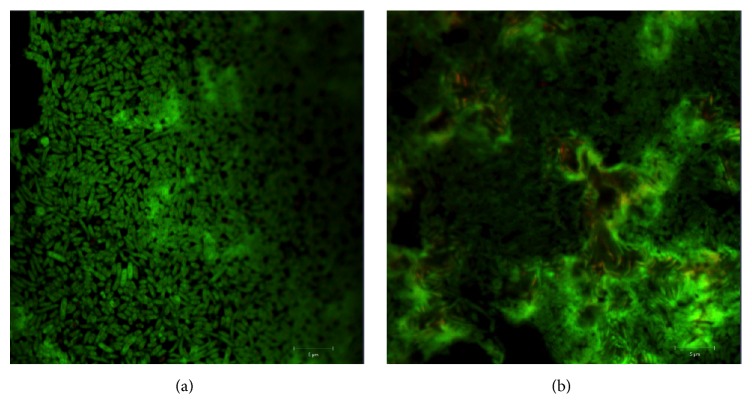
Fluorescent microscopy images of* S. marcescens* SR 41-8000 biofilm stained with DiOC_6_(3) and propidium iodide. Live and dead cells stained green and red, respectively. (a) 2-Day-old biofilm; (b) 4-day-old biofilm. Bars: 5 *μ*m.

CLSM showed that, after 2 days of growth,* S. marcescens *biofilm population consisted of individual cells with several microcolonies (Figure 3(a)). At this time, hardly any dead cells were detected. In contrast, 4-day-old biofilms consisted predominantly of microcolonies. The overall structure of the growing microbial biofilm became increasingly complex and multilayered. PI staining indicated the presence of the dead cells fraction in the developing biofilms (Figure 3(b)). These results are not surprising, since the cell death is a normal phenomenon during the biofilm development. The dead biomass constitutes an essential part of every biofilm and evidences that biofilm is a complex ever-changing dynamic system [37].

### 3.3. Biofilm Formation of the* S. marcescens* SR 41-8000 Reaches Its Maximum after Seven Days of Growth

**Figure 4:**
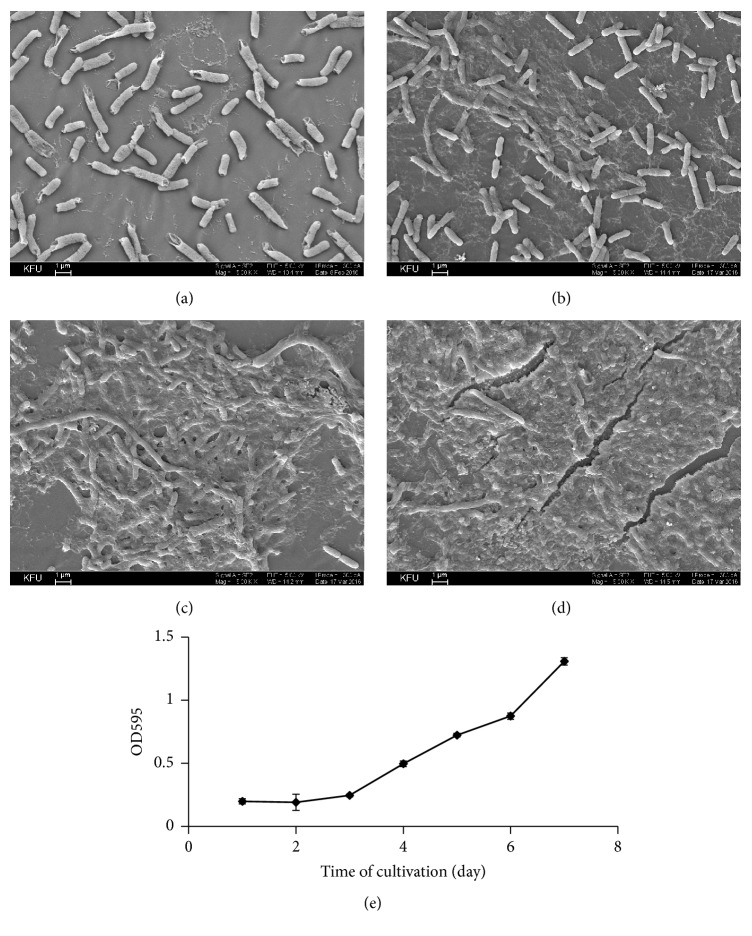
Time-course of the biofilm formation by* S. marcescens *SR 41-8000. Biofilm formation on catheters' surface was evaluated by SEM ((a)–(d)) after 1 (a), 3 (b), 5 (c), and 7 (d) days of cultivation in MH broth. Images were taken at 5,000x magnification. Bars: 1 *μ*m. Quantitative analysis of biofilm formation in crystal violet assay (e) was performed over 7 days of growth as described in Section 2. Experiments were performed in triplicate and repeated twice. Error bars represent standard deviations.

To establish a model for studying the efficacy of* Bacillus* proteolytic enzymes application in the eradication of mature biofilms, we performed a time-course experiment.* S. marcescens* SR 41-8000 cells were allowed to form biofilms on the catheters' surface incubated in the MH broth over 7 days. For in-depth microscopic analysis, catheters were visualized by SEM. We noticed that, during the first 2 days of growth, bacterial cells adhere to the surface of the catheter but still demonstrate a planktonic behavior. Contours of the cells can be easily defined, and there is no evidence of any cell-to-cell interactions (Figure 4(a)). After 3 days of cultivation, first changes in the morphology can be detected: chains of cells become visible; cells start to produce EPS that contribute to the development of a slimy biofilm layer (Figure 4(b)). Furthermore, after 5 days, the biofilm development continues, resulting in the formation of a mucoid and robust structure of the extracellular matrix. Well-developed microcolonies, 40 *μ*m in diameter, can be observed as tightly adherent on the surface. Cells are surrounded by a large amount of extracellular structural components of the biofilm matrix and appear to be in a close contact to each other (Figure 4(c)). Finally, after 7 days of growth, biofilms maturate and could be described as a relatively thick multilayered mat of bacterial cells embedded in a self-produced slimy matrix of the hydrated EPS (Figure 4(d)).

To quantify the amount of biofilms produced at different stages of development, we followed* S. marcescen*s biofilm production in MH broth over the course of 7 days. In agreement with SEM observations (Figures 4(a)–4(d)), first signs of the biofilm formation were observed after 4 days of growth. Biofilm production continued to increase over time and reached its maximum after 7 days of growth (Figure 4(e)). No further increase in the biofilm production was observed beyond that (data not shown).

### 3.4. *S. marcescens* SR 41-8000 Cells Produce Amyloid Fibrils

**Figure 5:**
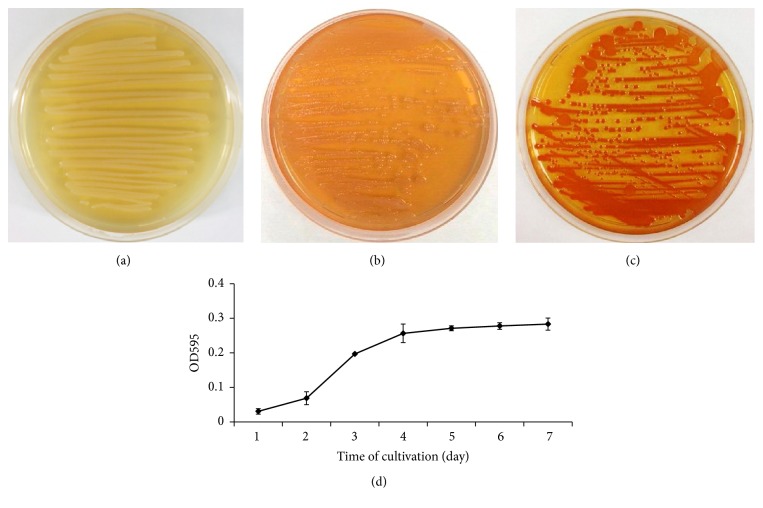
*S. marcescens* SR 41-8000 cells produce amyloid fibrils that absorb Congo red. (a) Bacteria grown on LB agar plate at 30°C for 3 days. ((b) and (c)) Bacteria grown on LB agar plates in the presence of CR dye (25 *μ*g/ml) at 30°C for 24 h (b) and 72 h (c). (d) CR absorption increases over the time in a linear fashion. Experiment was performed in triplicate and repeated twice. Each bar represents the average of three experiments, with error bars representing standard deviations.

Biofilm matrix serves as a protective barrier that prevents penetration of antimicrobial agents and bacteria killing. A lot of attention is being paid now to the proteinaceous matrix content, the amyloid-like fibrils of which have been demonstrated to play an important functional role in the bacterial colonization on the surface [10]. The amyloid-like fibrils appear to be a major component of the extracellular material, produced by both Gram-negative and Gram-positive bacteria that confer biofilm matrix integrity and stability [38]. Amyloid fibrils production by* S. marcescens* was examined in Congo red (CR) binding assay. On the 3rd day of cultivation, we observed the formation of red colonies that evidenced CR binding to the amyloid fibrils produced by* S. marcescens* (Figure 5(c)). Quantitative CR absorption experiments revealed a significant increase in the amount of amyloid fibrils on the 3rd day of cultivation. The production of amyloid fibrils continues to increase over the time of cultivation (Figure 5(d)).

### 3.5. *Bacillus* Proteases Efficiently Destroy* S. marcescens* Biofilms

There is a growing need for novel strategies aimed at the degradation of the biofilm protein components. Microbial proteases are one of the potential matrix-degrading agents that can be used in combination with antimicrobial agents for the total eradication of the biofilms. The rationale for these studies is that enzymatic degradation of the biofilm matrix is expected to inevitably lead to the disruption of the EPS and detachment of cells from the surface [12].

**Figure 6:**
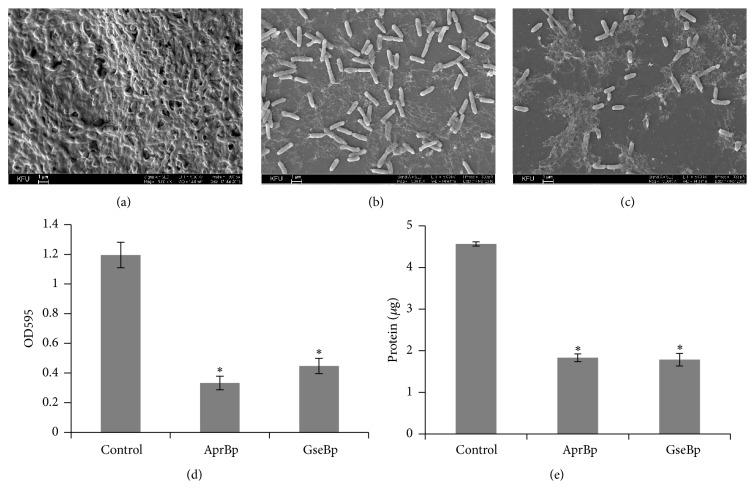
*Bacillus* proteases effectively destroy* S. marcescens *biofilms. ((a)–(c)) SEM micrographs illustrating* S. marcescens* biofilms degradation by* Bacillus* proteolytic enzymes. (a) Nontreated biofilms (biofilms incubated with 0.05 M Tris-HCl buffer). (b) Biofilms treated with subtilisin-like protease at 0.5 U/ml. (c) Biofilms treated with glutamyl endopeptidase at 0.1 U/ml. Images were taken at 5,000x magnification. Bars: 1 *μ*m. (d) Quantitative analysis of the 7-day-old* S. marcescens* biofilms' biomass after the protease treatment. Established biofilms were treated with subtilisin-like protease at 0.5 U/ml and glutamyl endopeptidase at 0.1 U/ml for 12 h at 37°C. Biofilms treated with 0.05 M Tris-HCl, pH 8.5, were used as a control. Experiment was performed in triplicate and repeated twice. Each bar represents the average of three experiments, with error bars representing the standard deviations. (e) Protein concentration in the EPS extracted from the protease-treated and untreated* S. marcescens *biofilms. Protein concentrations in the collected EPS extracts were quantified in Bradford assay. Experiment was performed in duplicate and repeated twice. Each bar represents the average of two experiments, with error bars representing standard deviations. Asterisk indicates statistical significance in Student's *t*-test with *p* < 0.05.

A high-yield extracellular production of proteolytic enzymes is one of the remarkable features of* Bacillus *species.* B. pumilus* 3-19 was found to be an important source of proteases such as subtilisin-like protease (AprBp) and glutamyl endopeptidase (GseBp) [30, 39]. Subtilisin-like protease, a 27 kDa extracellular protease, was purified from* B. subtilis* AJ73 recombinant strain carrying pCS9 plasmid with a gene encoding AprBp of* B. pumilus* 3-19 [40]. Glutamyl endopeptidase, a 23 kDa extracellular protease, was purified from* B. subtilis* JB 20-36 recombinant strain carrying Δ58.21 plasmid with a gene encoding GseBp of* B. pumilus* 3-19 [41]. Both proteases belong to a group of serine proteases and demonstrate high proteolytic and thrombolytic activity, suggesting their broad applications in medicine and industry. So far, it has been shown that AprBp and GseBp are capable of inducing fibrin lysis in blood clots and digesting aggregated *β*-amyloid peptides [42]. Here, we evaluated the potential of AprBp and GseBp to induce* S. marcescens* biofilms dispersal. Both enzymes were used to treat 7-day-old biofilms formed on the catheters. SEM analysis demonstrated that protease treatment resulted in a significant destruction of the biofilms compared to the untreated control. Microscopy of the biofilms treated with proteolytic enzymes revealed noticeable changes in their morphology. Protease treatment affected the EPS and led to the disintegration of the biofilm structure, detachment of cells from the catheter, and destruction of the numerous cell-to-cell contacts. Bacteria were no longer surrounded by a confluent mass of the EPS and only single cells were present (Figures 6(a)–6(c)). Quantitative crystal violet staining assay revealed that protease treatment resulted in a significant reduction of the* S. marcescens* biofilms' density. Glutamyl endopeptidase and subtilisin-like protease treatment destroyed 60–70% of the biofilm biomass (Figure 6(d)).

To confirm that protease treatment had a direct effect on the protein components of the extracellular matrix, we collected EPS from the protease-treated and untreated biofilms. Protease treatment with both enzymes (AprBp and GseBp) significantly reduced protein concentration in the resulting matrix (Figure 6(e)). Evidence supporting the concept that biofilms can be efficiently destroyed by proteases and nucleases has been illustrated by several investigators. The studies on* Bacillus* biofilms support the hypothesis that serine proteases are sufficient alone to remove biofilms by targeting key proteins engaged in the bacterial aggregation [43]. A more recent study demonstrated a significant reduction of the* B. licheniformis* biofilm after DNase I and Proteinase K (PK) treatment. The observations revealed that 64% of the amyloid-like proteins could be detached from the biofilm by enzymatic treatment. Interestingly, after either DNase I or PK treatment, a simultaneous removal of the extracellular DNA (eDNA) and extracellular proteins including amyloid fibrils (AF) was always observed, suggesting that these biofilm matrix components are interconnected with each other through intermolecular linkages [10]. The aggregative mechanism based on the eDNA and AF interactions has already been reported for many bacterial biofilms. For* Haemophilus influenzae*, it has been shown that type IV pilin protein and a significant amount of double-stranded DNA form together a dense intertwined meshwork providing structural biofilm stability [44]. Another study has demonstrated the formation of tightly associated amyloid-DNA complexes through DNA incorporation into the amyloid fibrils [45]. These observations suggest the high potential of the proteases for the biofilm removal, as long as the destruction of the closely interlaced matrix components, where proteins seem to play a crucial role, would collapse the whole structure of the biofilm.

Therefore, both enzymes, subtilisin-like protease and glutamyl endopeptidase, were found to be very effective in the biofilm removal through weakening the EPS integrity, which caused a subsequent detachment of the bacterial cells from the catheter. Our data support the idea that microbial proteolytic enzymes can be used as matrix-degrading agents. Several microbial proteases have already been implicated in the biofilm matrix degradation. Thus, purified ESP protease produced by* S. epidermidis* was found to suppress biofilm formation and nasal colonization by human pathogen* S. aureus* [14].* S. marcescens* serratiopeptidase enhanced the susceptibility of* P. aeruginosa* and* S. epidermidis* biofilms to ofloxacin [46] and inhibited the biofilm formation by* Listeria monocytogenes* [16]. Our study added two* Bacillus* proteases to the list of potent antibiofilm agents. The interest of* Bacillus* serine proteases AprBp and GseBp lies in the fact that these enzymes were able to disrupt tightly packed multilayered structure of the biofilms at very low concentrations. Being produced by nonpathogenic* B. subtilis* recombinant strain under the normal growing conditions, AprBp and GseBp can be rapidly purified and obtained in large quantities. Their cheapness and compatibility allow us to argue that these proteases can serve as a novel powerful tool for the biofilm eradication in both medicine and industry. Protease treatment of the biofilms may be predominantly used in medical practice to increase access to the bacterial cells and thereby enhance antibiotics efficacy in the therapy of bacterial infections.

## 4. Conclusion

In this study, we demonstrated that* Bacillus* proteolytic enzymes could be used as potential therapeutic agents to eradicate biofilms through the destruction of the biofilm matrix integrity. The use of subtilisin-like protease and glutamyl endopeptidase resulted in a significant decrease of the established biofilm biomass.
